# Complete diaphysis resorption of the femur: A case report in a metastatic papillary thyroid cancer

**DOI:** 10.1016/j.amsu.2020.11.076

**Published:** 2020-11-28

**Authors:** Suwardjo Suwardjo, Widya Surya Avanti, Ery Kus Dwianingsih, Wirsma Arif Harahap, Sumadi Lukman Anwar

**Affiliations:** a Division of Surgical Oncology, Department of Surgery, Dr Sardjito Hospital, Faculty of Medicine, Public Health, and Nursing, Universitas Gadjah Mada, Yogyakarta, 55281, Indonesia; b Department of Radiology, Dr Sardjito Hospital, Faculty of Medicine, Public Health, and Nursing, Universitas Gadjah Mada, Yogyakarta, 55281, Indonesia; c Department of Anatomical Pathology, Dr Sardjito Hospital, Faculty of Medicine, Public Health, and Nursing, Universitas Gadjah Mada, Yogyakarta, 55281, Indonesia; d Division of Surgical Oncology, Department of Surgery, Dr M Jamil Hospital, Faculty of Medicine Universitas Andalas, Padang, 25127, Indonesia

**Keywords:** Papillary thyroid cancer, Bone resorption, Delayed diagnosis, Metastasis, CTscan, Computed Tomography scan, TSH, Thyroid Stimulating Hormone, WHO, World Health Organization

## Abstract

**Introduction:**

Although differentiated thyroid cancers generally have a good prognosis, a small proportion of patients will have recurrent or progressive disease. Bone resorption due to thyroid cancer can cause significant challenges in the clinical management and rehabilitation.

**Presented case:**

Nearly total femur resorption was found as a first presentation in a patient with thyroid cancer. The patient complained about chronic pain in her left thigh that had progressed into an inability to walk. She was treated by a traditional healer for six years before she was persuaded by a social worker to seek medical help. X-rays showed pathological loss of the right diaphyseal femur. Neck CT-scan showed a left thyroid mass with tracheal deviation, with multiple lytic lesions in the sternum and 5th rib. Needle biopsy of the thyroid mass resulted in an inconclusive follicular neoplasm. Total thyroidectomy and neck dissection revealed a classical type of papillary thyroid carcinoma. After thyroid ablation, she opted for palliative radiotherapy and bisphosphonate treatment for the bone metastases.

**Discussion:**

Bone metastases are rarely detected at the time of thyroid cancer diagnosis. In the presence of bone metastasis, median survival of well-differentiated thyroid cancer decreases into only 4 years. Bone metastases are often neglected and less studied than regional lymph node and lung metastases.

**Conclusion:**

Although well differentiated thyroid cancer is usually indolent, a neglected bone metastasis at an initial diagnosis might adversely affect patient's quality of life and prognosis.

## Highlights

• Although differentiated thyroid cancer is usually indolent, delayed diagnosis might adversely affect the outcome.
• Macro-metastases in the bone might cause poor functioning and prognosis.
• Public education about high-risk thyroid cancer is important to prevent from neglected cases.

## 1 Introduction

Thyroid cancer is the fifth most common cancer among females worldwide and the sixth in Indonesia [1,2]. Every year, around 440,000 women including 8000 women from Indonesia are diagnosed with thyroid cancer [1,2]. Although the 5-year survival rate is above 95%, the overall survival rates vary greatly according to specific types of thyroid cancer and the stage at diagnosis [3]. Thyroid cancers infrequently cause distant metastases and manifestation in the bones is found only in 4% [3]. Bone metastasis are independently correlated with worse prognosis with median survival rate of only four years after the detection [4].

The recent controversy of thyroid cancer management at the population level concerns the effectiveness of early detection and active surveillance [5]. Incidence of thyroid cancer has tripled in the past 40 years although the mortality rates remain steadily low suggesting the excess detection of subclinical diseases [5]. Mass screening and early detection might cause overdiagnosis of naturally benign or low-risk thyroid cancer [5,6]. However, delayed diagnosis and treatment initiation that often occur in developing countries might affect patient's long-term outcome and functioning. Identification and stratification of high-risk papillary thyroid cancer are required to determine suitable treatment and surveillance. To describe the impacts of low cancer awareness, delayed diagnosis, and the challenges of treatments in metastatic thyroid cancer, we reported an unusual case of nearly complete resorption of the left femur as a metastatic manifestation of classical papillary thyroid cancer. The presented case was reported in accordance with the SCARE 2018 principles [7].

## 2 Presentation of case

**Fig. 1:**
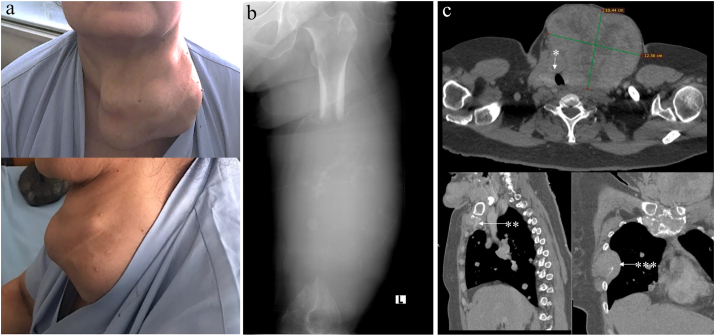
(a) A large multinodular neck mass was observed in a – 51 years old woman who presented in an oncology clinic with a gross deformity of the left side. (b) An expanded destructive lytic lesion in the left femur causing almost complete loss of the diaphysis was shown in X-ray. Some remaining bone fragments were observed within a large swelling of the surrounding soft tissues of the eroded bone. (c) Cervical contrast CT-scan showed a large thyroid mass of 12.5 cm in the diameter with tracheal deviation and 30% lumen narrowing (*). Thoracic CT-scan showed multiple lytic lesions in the manubrial sternum (**) and destructed 5th rib with expansive swelling (***).

A 51-year-old woman was referred to the oncology unit with pathological fracture of the left femur and a prominent neck mass. She complained about intermittent chronic pain in the right thigh which worsened at night and was relieved with movement during the day. After seeking treatment from a traditional healer for six years in which she received massage, stretching, and herbal medicine, she finally sought medical help after becoming unable to walk. She was accompanied by a social worker who encouraged her to undergo medical treatment. Thorough history-taking revealed that the patient had slowly grown the neck mass in the last 20 years without any complaint of palpitations, agitation, sweating, loss of weight, dysphagia, hoarseness, or dyspnea. Any medical history and hospital admission were denied. No family history of thyroid problems nor endocrine cancer were reported. The patient resided in a remote rural area and had not graduated from an elementary school. The patient did not have any complaint about the large neck lump because there was no direct effect to her daily activities. She believed that her health problem was mainly in her left leg after she accidently fell in her bedroom and opted to receive traditional treatments from a bone healer only. A multinodular thyroid mass with approximately 14 cm in diameter was palpated in the neck with observed firm consistency, ill-defined borders, pushing the trachea to the contralateral, and moving during swallowing (Fig. 1a). We then performed neck CT-scan, sonography, and needle biopsy to confirm the primary origins of the cancer from the thyroid (Fig. 1c). Soft tissue swelling with soft consistency and unclear borders was observed in the left mid-femur with diameter of 20 cm. Limited movement with a non-functional left leg were observed. She could not move the left leg including flexion-extension, adduction-abduction of the knee; dorsi-plantar flexion, eversion and inversion of the ankle; and plantar-dorsiflexion of the toes. Sensory modalities were intact with slightly impaired modalities in the middle thigh for light touch.

The differential diagnosis was metastatic thyroid cancer either from differentiated or undifferentiated carcinoma. Although relatively rare, thyroid lymphoma or metastatic manifestation of the soft tissue cancer from the femur might be considered. The swelling in the mid shaft of the femur was also differentially diagnosed with a transformed sarcoma from Paget's disease and primary bone tumors. Metastatic manifestation in the femur might cause permanent loss of the left leg and decrease quality of life due to the potential infiltration to the femoral nerve. However, the primary thyroid cancer was potentially able to be completely resected with total thyroidectomy accompanied by modified radical neck dissection. Extrathyroidal extension to the surrounding tissues might hinder attempt for complete resection of the primary thyroid cancer.

**Fig. 2:**
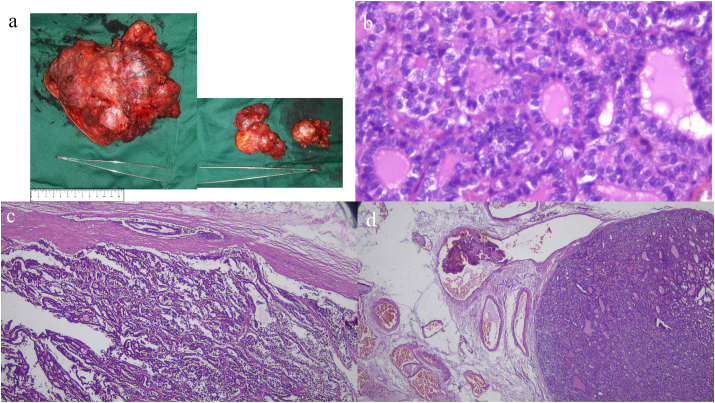
Macroscopically, the tumor from the left lobe was around 17 cm in diameter with irregular multinodular lesions and intra-capsular grey to white tumor (a). The tumor from the right thyroid lobe was 6 cm in diameter and enlargement of multiple lymph nodes (range 0.8–3.2 cm in diameter, panel a). Polymorphic and enlarged tumor cells with round nuclei, coarse chromatin, prominent nucleoli forming a ground glass appearance (b). Infiltration to thyroid capsule (c), veins (b), lymphatic vessels, surrounding skeletal muscles and soft tissues were also observed.

The total thyroidectomy and modified lymph node dissection were performed by a surgical team consisted of experienced and national board-certified oncological surgeons in the management of advanced thyroid cancer. Pathological examination after surgery confirmed a classical type of papillary thyroid carcinoma with capsular and lympho-vascular infiltrations. Largest diameter of the multifocal tumor was 17 cm, and 8 lymph nodes were infiltrated by the cancer cells (Fig. 2). Thyroid ablation was performed twice i.e., two months after surgery and six months later with total accumulative dose of I-131 200mCi. Patient was treated with levothyroxine to maintain TSH level below 0.1 μIU/mL. Because the left leg was not functional, limb salvage was offered. However, she opted for palliative radiotherapy and bisphosphonate treatment for the bone metastasis. External beam radiotherapy of 20 Gy in 5 fractions and monthly zoledronic acid were administrated to the patient.

She has recovered well after one-year follow-up from the initial diagnosis. Monthly physical examination, three-monthly sonography, and biannually laboratory and neck imaging, were performed in the first-year hospital-based surveillance program. The thyroid cancer was controlled with no evidence of locoregional progression and no new sites of distant metastasis. She continued with TSH suppression therapy. Although her left leg was still not functional, in the second offering, she decided not to have limb salvage surgery. While confined to the wheelchair, she could still complete her daily living activities independently.

## 3 Discussion

Epidemiological studies have revealed a triple increased rate of thyroid cancer during the past four decades worldwide [5,8]. The steep increase of thyroid cancer incidence is associated with the current ability to detect small and subclinical thyroid cancer using available imaging techniques [5,8]. Early detection and health surveillance for thyroid cancer in asymptomatic individuals have also been associated overdiagnosis and overtreatment [5]. However, for symptomatic individuals particularly with thyroid nodules larger than 1 cm, thorough anamnesis and physical examination followed with sonography, cytology assessment, and risk stratification are recommended.

According to the histological patterns, thyroid cancers are classified into differentiated carcinoma consisting of papillary and follicular carcinoma and undifferentiated anaplastic carcinoma [3]. Thyroid cancer originating from parafollicular cells is known as medullary carcinoma [3]. Papillary carcinoma is the most common subtypes comprising 80–85% of all thyroid malignancy [9]. Papillary thyroid carcinomas are commonly diagnosed in the age of 30–50 years old and affect more women than men (2- to 4-fold higher rate) [3,9]. Macroscopically, papillary thyroid cancers vary in size (average 2.5 cm) and are usually firm, white in color, with existence of calcification [9]. Microscopically, papillary thyroid carcinomas are characterized with enlarged clear nuclei containing hypodense chromatin known as ground glass appearance [9]. The classical papillary type is identified with formation of neoplastic cells into complex branching and irregular formation of papillae surrounded by fibrovascular cores, as shown in our case (Fig. 2). Invasion to the lymphatic system is relatively common in papillary thyroid carcinoma often causing intraglandular multifocality [3,9]. Metastases to cervical lymph nodes particularly to level VI (central) are found in more than 50% papillary thyroid cancer at initial presentation. Tumor infiltration to central lymph nodes does not adversely affect overall prognosis particularly for patients under 55 years old [9]. However, classic papillary thyroid carcinoma rarely invades venous and capillary systems [9,10]. Distant metastasis of papillary thyroid carcinoma is usually found after long term follow-up (more than 10 years) and was reported in 5–7% patients in which 4% of them were manifested in the bone [3]. Distant metastasis at initial diagnosis is very rarely found in papillary thyroid cancer [3].

Treatment and surveillance of patients with papillary thyroid cancers are delivered according to the risk stratification following the World Health Organization (WHO) criteria [3]. Tumor diameter more than 4 cm, lympho-vascular invasion, capsular invasion, macroscopic invasion to peri-thyroid soft tissues, pathological N1 disease and one or more nodal size larger than 3 cm, with distant metastasis as found in our case are classified as a high-risk papillary thyroid cancer [3]. In high-risk papillary cancer cases, total thyroidectomy and neck dissection followed by radioisotope ablation are recommended [3,11]. For patients with distant metastasis, it is suggested to give adjuvant high dose thyroid ablation up to 200 mCi after levothyroxine withdrawal [12]. If the distant metastases absorb I-131, the thyroid ablation can be performed every 6 months for 2 years to reach a cumulative dose of 600 mCi [9]. Levothyroxine suppression should be given in between ablation to suppress TSH levels below 0.1 mIU/mL. Maintaining TSH suppression during long-term follow-up is associated with lower disease progression, recurrence and mortality rates [3,12].

Skeletal-related events, as in our case were presented as pathological fracture, nearly total resorption of the femur, and lytic lesions in the sternum and costae, which are associated with poorer prognosis. Bone metastases are more commonly found in follicular than papillary thyroid cancer. The predilection sites are in the spine (34.6%) and pelvic bones (25.5%) whereas the thoracic bones and extremities are rare comprising 18.3% and 10.2% of total bone metastases, respectively [4]. Bone metastases are commonly found accidently from laboratory or imaging findings. In our case, we also observed soft tissue swelling around the bone resorption as a manifestation of distant metastasis. Repeated injury or massaging might cause chronic inflammation that would further facilitate the formation of a metastatic niche for the metastatic cells to grow [13]. In the presence of bone metastases, bone remodeling treatment using bisphosphonates and external beam radiotherapy are recommended [3,12]. High-dose thyroid ablation is also able to control the progression of metastasis and to alleviate the symptoms. In oligometastatic bone manifestation, locoregional treatment can promote better progression-free survival or cure [12,14]. Surgery followed by radiotherapy usually result in better outcome particularly for bone lesions in the limbs [12,14]. If surgery is not possible, radiotherapy for 20–30 Gy in 5–10 fractions is recommended [12]. Palliative radiotherapy can relieve pain and other neurological complications although the effects are usually attained 2–3 days after the treatment.

Unusual manifestations of differentiated thyroid carcinoma have been reported as labia majora swelling, femur neck lesion, iliac crest swelling, as well as skin and pancreatic lesions as summarized by Iftikhar et al. [15]. Brain and skull metastases are also rarely reported from well-differentiated thyroid cancer and are often associated with worse prognosis [16]. Survivorship and quality of life are severely affected in patients with thyroid cancer particularly in the presence of distant metastases [17]. Our report elaborated the unique case of bone macro-metastasis with a skeletal event at an initial diagnosis in a high-risk classical papillary thyroid carcinoma after a significantly delayed diagnosis.

The majority of differentiated thyroid cancers are regarded as indolent tumors [5,10]. However, identification of patients with high-risk and advanced stage are very important to prevent over-diagnosis and over-treatment in those with low-risk disease or benign nodules. Our case reflected the complexity of cancer management with the nature of an indolent cancer that was presented in metastatic stage after a significant delayed of diagnosis. There are widespread challenges for cancer treatment in developing countries [8,18,19] in which cancer patients are mostly diagnosed in advanced stages and with metastatic disease. Regarding thyroid cancer, public education might be needed to raise awareness of high-risk thyroid cancer to reduce delayed diagnosis and treatment. In addition, improving associated sociodemographic factors that contribute to the potential delayed diagnosis of thyroid cancer is required. Misunderstanding about cancer, fear of disease and treatment, non-attribution of symptoms to the primary cancer, and low educational level are among the most frequent causes of patient associated delayed cancer diagnosis that should be further addressed.

## 4 Conclusion

Despite well-differentiated thyroid cancer is generally associated with good prognosis, a neglected bone metastasis at an initial diagnosis could adversely affect patient's quality of life and prognosis. Public education for high-risk thyroid cancer is required to prevent delayed diagnosis, metastatic disease at first presentation, and deterioration of quality of life.

## Sources of funding

SLA received Dana Masyarakat (133/2020) and RTA (2488/2020) grants from 10.13039/501100012521UGM, and NUS-UGM-Tahir Foundation seed grant (1/2020).

## Ethics approval

Not applicable.

## Consent for publication

Written informed consent was obtained from the patient for reporting the case and displaying the relevant images. De-identification of images and related materials were used in this manuscript. A copy of the written informed consent is available for review by the Editor-in-Chief of this journal on request.

## Authors’ contributions

SS, WAH, SLA conceptualized the report and finalized the manuscript. EKD provided and gave expertise in the tumor histopathology. WSA gave expertise in the imaging. SS and SLA were involved in the surgery and care of the patient. All authors read and approved the final manuscript.

## Registration of research study

Not applicable.

## Guarantor

SLA.

## Provenance and peer review

Not commissioned, externally peer reviewed.

## Availability of data and materials

The clinical and imaging data supporting the analysis and findings of this study will be available from the corresponding author upon reasonable request.

## Declaration of competing interest

No potential competing interest has been declared from all authors.
